# Identification of Novel HLA-A*24:02-Restricted Epitope Derived from a Homeobox Protein Expressed in Hematological Malignancies

**DOI:** 10.1371/journal.pone.0146371

**Published:** 2016-01-19

**Authors:** Maiko Matsushita, Yohei Otsuka, Naoya Tsutsumida, Chiaki Tanaka, Akane Uchiumi, Koji Ozawa, Takuma Suzuki, Daiju Ichikawa, Hiroyuki Aburatani, Shinichiro Okamoto, Yutaka Kawakami, Yutaka Hattori

**Affiliations:** 1 Division of Clinical Physiology and Therapeutics, Keio University, Faculty of Pharmacy, Tokyo, Japan; 2 Division of Pharmacy, National Cancer Center Hospital, Tokyo, Japan; 3 Genome Science Laboratory Research Center for Advanced Science and Technology, The University of Tokyo, Tokyo, Japan; 4 Division of Hematology, Keio University, School of Medicine, Tokyo, Japan; 5 Division of Cellular Signaling, Institute for Advanced Medical Research, Keio University, School of Medicine, Tokyo, Japan; Baylor College of Medicine, UNITED STATES

## Abstract

The homeobox protein, PEPP2 (RHOXF2), has been suggested as a cancer/testis (CT) antigen based on its expression pattern. However, the peptide epitope of PEPP2 that is recognized by cytotoxic T cells (CTLs) is unknown. In this study, we revealed that *PEPP2* gene was highly expressed in myeloid leukemia cells and some other hematological malignancies. This gene was also expressed in leukemic stem-like cells. We next identified the first reported epitope peptide (PEPP2^271-279^). The CTLs induced by PEPP2^271-279^ recognized PEPP2-positive target cells in an HLA-A*24:02-restricted manner. We also found that a demethylating agent, 5-aza-2’-deoxycytidine, could enhance PEPP2 expression in leukemia cells but not in blood mononuclear cells from healthy donors. The cytotoxic activity of anti-PEPP2 CTL against leukemic cells treated with 5-aza-2’-deoxycytidine was higher than that directed against untreated cells. These results suggest a clinical rationale that combined treatment with this novel antigen-specific immunotherapy together with demethylating agents might be effective in therapy-resistant myeloid leukemia patients.

## Introduction

The development of chemotherapy regimens and targeted therapies has improved the survival of patients with leukemia. However, this disease continues to recur following conventional therapies in some patients, leading to poor prognosis [1,2]. Therefore, eradication of residual disease by additional treatment is necessary for these therapy-resistant patients. By this rationale, immunotherapy using antigen specifically expressed by leukemic cells might be an attractive strategy to cure leukemia patients [3,4,5].

Several leukemia-associated antigens (LAAs) have been reported, which include Wilms’ tumor 1 (WT1), proteinase-3, bcr-abl or PML-RARα[3,6]. Some of these are now in clinical trials [7,8,9]. However, their clinical benefits remain to be proven in some cases, although these LAAs can elicit antigen-specific immune responses in patients. One of the reasons for this discrepancy might be that recognition of only one antigen is not sufficient to eradicate leukemic cells in patients, because cancer cells could escape the immune response in various ways including down-regulating antigens. Recent studies have shown that using multiple epitopes in a vaccine setting is more effective than using a single peptide, therefore, identification of novel LAAs is necessary to enhance anti-tumor effects [10,11].

Another key to improving the efficacy of LAA-targeted immunotherapy is to choose LAAs expressed by leukemic stem cells (LSCs). Evidence suggests that LSCs are resistant to chemotherapy or targeted therapy such as tyrosine kinase inhibitors by several mechanisms, including maintenance of a resting state or the expression of a high number of drug-efflux pumps [12,13,14]. Immunotherapy targeting antigens expressed by LSCs would not be affected by these characteristics of LSCs and, therefore, could eliminate these cells.

PEPP2, which is also called RHOXF2, was first identified by Wayne et al [15] as a homologue of the mouse *Pem1* gene, which belongs to the homeobox genes regulating sperm differentiation. This gene has been suggested to be a cancer/testis antigen (CTA) based on its expression pattern [16,17]. Cancer/testis antigens are known to be good therapeutic targets, since their expression in normal tissue is limited to testis, which is an immune-privileged site lacking HLA expression [18]. However, a PEPP2-derived epitope recognized by cytotoxic T lymphocytes (CTLs) has not yet been identified. Herein, we report the successful identification of the first CTL epitope derived from PEPP2. Using this epitope, we could induce CTLs that targeted PEPP2-positive cancer cells.

It has also been reported that gene expression of many CTAs is controlled by methylation of CpG islands in their promoter regions [18]. Therefore, demethylating agents could up-regulate the expression level of these antigens. In this study, we also investigated the effects of demethylating agent on PEPP2 expression and recognition by CTLs.

## Materials and Methods

### Cell lines

Cancer cell lines K562, KU812 (CML), KG1a, NB4, HL60 and PL21 (AML), HEL (erythroleukemia), U937 (histiocytic leukemia), BALL1 (acute lymphoblastic leukemia), Molt4 (acute lymphoblastic leukemia), Daudi, Sultan (Burkitt’s lymphoma) were obtained from the Health Science Research Resources Bank (National Institute of Biomedical Innovation, Osaka, Japan). The KMS11, KMS20, KMS21, KMS26, KMS28 and KMS34 cell lines were kindly gifted by Dr. Ohtsuki of Kawasaki Medical School (Kurashiki, Japan). These cells were maintained at 37°C in a humidified atmosphere of 5% CO_2_ in RPMI-1640 medium (Sigma-Aldrich, Saint Louis, MO) and 10% fetal bovine serum (Invitrogen^™^, Life Technologies, Grand Island, NY)

### Patient samples

Peripheral blood samples from healthy donors (HD) and leukemic patients and bone marrow (BM) samples from leukemia patients were obtained after informed consent. Mononuclear cells were isolated by Ficoll density gradient centrifugation using Lymphoprep (NYCOED, Oslo, Norway) and cryopreserved in Cell Banker reagent (Juji-Field, Tokyo, Japan) in liquid nitrogen until further use. Bone marrow mononuclear cells (BMMNC) from healthy donors were obtained from AllCells, LLC. (Alameda, CA). This study was approved by the ethics committee of the Keio University School of Medicine (13-67-5) and Faculty of Pharmacy (111004–1). Written informed consent was obtained from all patients.

### Reagents

Paraformaldehayde (Wako-Jyunyaku, Tokyo, Japan),Triton X (Sigma-Aldrich), and bovine serum albumin (Sigma-Aldrich) were used for immunohistochemical staining. Phtohemagglutinin (Sigma-Aldrich) was used for generating PHA blasts in CTL induction. 5-aza-2′-deoxycytidine (Sigma-Aldrich) was dissolved in DMSO (Sigma-Aldrich) and diluted with RPMI-1640 medium (Sigma-Aldrich) before use.

### Sorting of LSCs

Leukemia cells were washed with phosphate-buffered saline (PBS; Sigma-Aldrich) and stained with R-phycoerythrin (PE)-conjugated monoclonal antibody against CD34 and fluorescein isothiocyanate (FITC)-conjugated monoclonal antibody against CD38 (Miltenyi Biotec, Auburn, CA) for 30 minutes on ice. After washing with PBS, cells were analyzed and separated using a MoFlo cell sorter system (Beckman Coulter Inc., Brea, CA).

### Detection of *PEPP2* expression with RT-PCR

Expression of the *PEPP2* gene was detected by standard RT-PCR or quantitative PCR. Total RNA was extracted from cancer cell lines, BMMNC from patients or HD, and PBMNC from HD using Isogen (Nippon Gene Co. Ltd, Tokyo, Japan). Testis RNA was purchased from BD Biosciences (Palo Alto, CA). Complementary DNA was synthesized from 1 μg of the total RNA using GoScript Reverse Transcription System (Promega, Madison, WI), followed by PCRs with PEPP2 primers (Forward; 5′-ggcaagaagcatgaatgtga-3′, Reverse; 5′-ggctgtggtcccagaagtaa-3′) or GAPDH primers (Forward; 5′-tgaacgggaagctcactgg-3′, Reverse; 5′-tccaccaccctgttgctgta-3′) with Taq polymerase (Takara Bio, Shiga, Japan). Quantitative PCR was performed using SsoFast probes supermix (Bio-Rad, Hercules, CA) with PEPP2 primers (Forward; 5′-gcagtgcagatttggtttgaga-3′, Reverse; 5′-tgccattaatgccctctgatg-3′) and probe (5′-(6-FAM)-tagaagagccaaatggagg-(BHQ-2)-3′) or GAPDH primers (Forward; 5′-gacctgacctgccgtctagaaa-3′, Reverse; 5′-cctgcttcaccaccttcttga-3′) and probe (5′-(6-FAM)-acctgccaaatatgatgac-(BHQ-2)-3′).

### Detection of *WT1* gene expression with RT-PCR

Expression of WT1 was detected in some of the APL patient bone marrow cells by standard PCRs using WT1-specific primers (Forward; 5′-ggcatctgagaccagtgagaa-3′, Reverse; 5′-gagagtcagacttgaaagcagt-3′) as previously described [19].

### Treatment with demethylating agent

PL21 and HL60 cells and PBMNCs from HD were cultured in AIM-V medium (Life Technologies) supplemented with 10% human serum (Lonza, Walkersville, MD) in 6-well culture plates and 200 nM of 5-aza-2′-deoxycytidine (Sigma-Aldrich) was added every 24 hours for 72 hours.

### Immunohistochemical staining

Immunohistochemical staining was performed as follows: Cells were attached to a slide using a Cytospin^™^ 4 cytocentrifuge (Thermo Fisher Scientific, Waltham, MA), then fixed with 2% paraformaldehyde and permeabilized with Triton X followed by blocking with 1% bovine serum albumin (BSA) in PBS. The sample was stained with monoclonal anti-PEPP2 antibody (Sigma-Aldrich) followed by Alexa Fluor488^®^ goat-anti-mouse IgG (Molecular Probes, Life Technologies)

### Synthetic Peptides

PEPP2-derived peptides which could be presented by HLA-A*24:02 were identified using the Bioinformatics and Molecular Analysis Section (BIMAS) program (http://www-bimas.cit.nih.gov/molbio/hla_bind/index.html), and the SYPFPEITHI program (http://www.syfpeithi.de/). HLA-A*24:02 is the most popular HLA-A type in Japanese population. These peptides were synthesized and purified to >95% by HPLC (Sigma-Aldrich Japan, Hokkaido, Japan). Purified peptides were dissolved in DMSO and stored in aliquots at -80°C. An HLA-A*24:02-binding peptide derived from Epstein-Barr virus (EBV; TYGPVFMCL) was used as a positive control.

### Peptide binding assay and peptide stability assay

We assessed the binding ability of candidate peptides to HLA-A24 as described previously [14]. CIR-A24 cells were incubated with 50 μg/mL peptides and 5μg/ mL of β2-microglobulin in serum-free media for 24 hours. After washing with PBS, the expression of HLA-A24 was measured by staining the cells with FITC-conjugated anti-HLA-A24 antibody (Mitsubishi-Kagaku, Tokyo, Japan). We also checked the stability of candidate peptides bound to the HLA-A24 molecule. CIR-A24 cells were incubated with 50 μg/mL peptides and 5 μg/mL of β2-microglobulin in serum-free media for 24 hours, followed by addition of 50 μg/ mL of brefeldin A (Sigma-Aldrich) for 4 hours. The expression of HLA-A24 was detected by staining the cells with FITC-conjugated anti-HLA-A24 antibody (Mitsubishi). MFI (mean fluorescence intensity) ratio was calculated as follows; MFI of CIR-A24 pulsed with each candidate peptide / MFI of CIR-A24 without peptide.

### Generation of PEPP2 peptide-specific CTLs from human PBMCs

CTLs were generated by *in vitro* stimulation with peptide-pulsed autologous dendritic cells and phytohemagglutinin (PHA) blasts. Briefly, PBMNCs were isolated from whole blood of HLA-A*24:02-positive HD by Ficoll density gradient centrifugation. Dendritic cells (DC) were generated by isolating CD14^+^ cells using a MACS separation system (Miltenyi Biotec, Bergisch Gladbach, Germany). The CD14^+^ cells were cultured in AIM-V medium supplemented with 10% human serum, 100 ng/mL of IL-4 (R&D Systems) and 100 ng/mL of GM-CSF (R&D Systems) for 5 days, then 20 ng/mL of TNF-α (R&D Systems) was added to generate DC. PHA blasts were derived from CD14^-^ cells by culturing in AIM-V medium supplemented with 10% human serum, 100 units of IL-2 and 1μ g/mL of PHA for 2 days. The DC or PHA blasts were pulsed with 50μg/L of peptide at room temperature for 3 hours and then irradiated (mediXtec, Chiba, Japan). On day 0, CD14^-^ cells were stimulated with irradiated peptide-pulsed DC in AIM-V medium with 10% human serum supplemented with 10 ng/mL of IL-7 (R&D Systems), then 50 ng/mL of IL-2 was added every 2 to 3 days. The cells were stimulated weekly with irradiated peptide-pulsed autologous PHA blasts, then CD3^+^CD8^+^ cells were purified with immunomagnetic beads (Miltenyi Biotec).

### Enzyme-linked immunospot (ELISpot) assay

IFN-γ secretion of CTLs in response to target cells was detected using ELISpot assay. Briefly, effector cells were incubated with target cells in plates coated with anti-IFNγ antibody (1-D1K; Mabtech Inc., Cincinnati, OH). After incubation for 20 hours, biotinylated antibody specific for IFN γ (7-B6-1; Mabtech) was added for 2 hours at room temperature and then streptavidin-alkaline phosphatase (Mabtech) was added for 1 hour. To develop spots, nitroblue tetrazolium and 5-bromo-4-chloro-3-indolyl phosphate (Biorad) were added and the color development was stopped by rinsing with distilled water. The resulting spots were counted using a CTL-ImmunoSpot^®^ analyzer (Cellular Technology Ltd., Shaker Heights, OH)

### Cytotoxicity assay

The cytotoxic activity of the CTLs was measured using a standard ^51^Cr release assay. Target cells were labeled with 50 μCi of ^51^Cr (PerkinElmer Inc., Waltham, MA) for 60 min at 37°C. CIR-A24 cells were then incubated with 5 μg/mL of peptide for 1 hour. For the blocking assay, KMS21 cells were treated with mouse anti-HLA class I antibody (w6/32; eBioscience, San Diego, CA) or mouse IgG isotype antibody (eBioscience). The target cells were mixed with effector cells. After 4 hours, supernatants were transferred to LumaPlates (PerkinElmer) and allowed to air dry in a hood overnight. Plates were sealed and counted in MicroBeta^®^ Trilux instrument (PerkinElmer). Percent specific lysis for ^51^Cr release was calculated as [(experimental ^51^Cr release—spontaneous ^51^Cr release) / (maximum ^51^Cr release—spontaneous ^51^Cr release)] x 100.

### Expression of HLA class I, HLA-DR, and PD-L1

Untreated cells or cells treated with 5-aza-2′-deoxycytidine were washed with PBS and stained with PE-conjugated monoclonal antibody against HLA class I (eBioscience), PE-conjugated monoclonal antibody against HLA-DR (Gen-Probe, San Diego, CA), PE-conjugated monoclonal antibody against PD-L1 (eBioscience) and mouse isotype IgG_1_ antibody (Beckman Coulter) for 30 minutes on ice. After washing with PBS, cells were analyzed using a FACSCalibur flow cytometer equipped with CellQuest software (BD Biosciences, San Jose, CA).

Expression of the *PD-L1* gene was detected by quantitative PCR. Extraction and cDNA synthesis from PL21 cells were conducted as described in ‘Detection of *PEPP2* expression with RT-PCR’ section. Quantitative PCR was performed using KOD SYBR qPCR Mix (TOYOBO, Osaka, Japan) with PD-L1-specific primers (Forward; 5-gacctatatgtggtagagtatggtagc-3′, Reverse; 5-tgccattaatgccctctgatg-3′). Quantitative PCR of *GAPDH* gene was performed as described above. The relative expression of PD-L1 was calculated by dividing results of PD-L1 by that of GAPDH.

### Statistical analysis

Results are presented as mean ± s.e. Groups were compared using Student’s *t*-test. Differences were considered significant when p < 0.05.

## Results

### *PEPP2* gene expression in cancer cells

**Fig 1:**
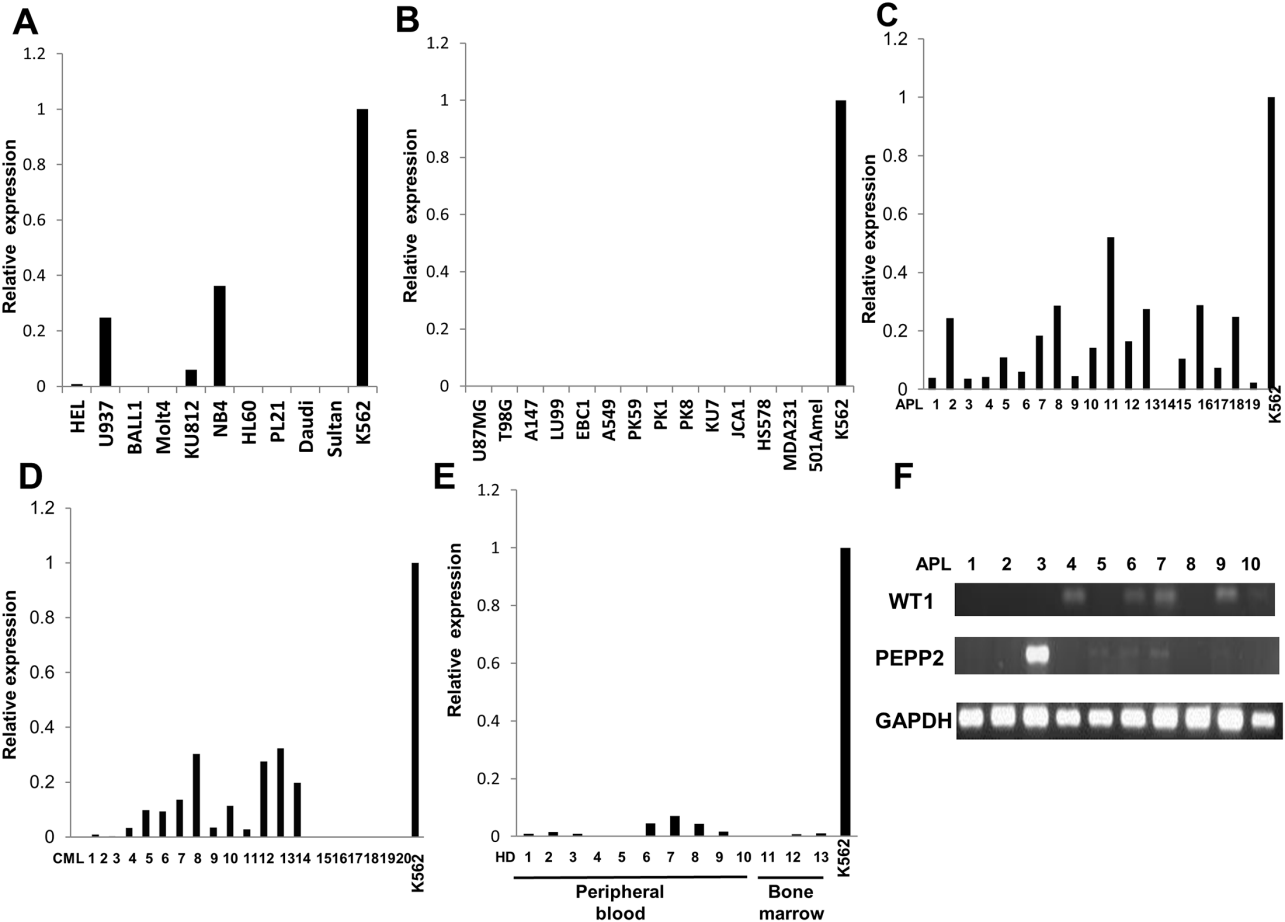
Expression profile of PEPP2 gene evaluated by quantitative RT-PCR. *PEPP2* gene expression was detected by quantitative RT-PCR. (A) Expression of *PEPP2* gene in cell lines of hematological malignancy. (B) Expression of *PEPP2* gene in other cancer cells. (C) Expression of *PEPP2* gene in bone marrow (BM) samples from APL. (D) Expression of *PEPP2* gene in BM samples from CML. (E) Expression of *PEPP2* gene in PBMNC or BMMNC from healthy donors (HD). (F) Expression of *PEPP2* gene and *WT1* gene was detected by standard RT-PCR. Relative expression was calculated by dividing expression level of PEPP2 by that of GAPDH. Quantitative PCR was conducted in triplicate. These figures show the representative data of 3 independent experiments.

We evaluated *PEPP2* gene expression in various types of cancer cell lines by quantitative RT-PCR (Fig 1A and 1B). Especially myeloid leukemia cells such as K562 (CML), NB4 (APL), KU812 (CML), and also U937 (histiocytic lymphoma) expressed the gene at high levels, which was compatible with the results of DNA microarray (A of S1 Fig). K562 expressed highest level of *PEPP2*. Expression level of testis was about 35% of K562 (0.347 vs. 1) (B, C of S1 Fig). Therefore, we decided to use K562 as a positive control. The expression of *PEPP2* was not observed in other types of cancer cell lines including U87MG, T98G, A147 (brain tumor), LU99, EBC1, A549 (lung cancer), PK59, PK1, PK8 (pancreatic cancer), KU7 (bladder cancer), JCA1 (prostate cancer), HSS78 (breast cancer), MDA231 (renal cancer), and 501Amel (melanoma).

We also examined *PEPP2* gene expression in leukemic patients’ samples. High expression was observed in 11 of 19 BM samples from APL (Fig 1C) and 8 of 20 CML patients (Fig 1D). On the other hand, PBMNC and BMMNC isolated from healthy donors expressed little to no levels of the gene (Fig 1E). In addition, the *PEPP2* gene expression pattern did not fully correlate with that of the *WT1* gene in APL samples. For example, APL sample 3 and 5 were *PEPP2*-positive and *WT1*-negative (Fig 1F).

### PEPP2 expression in leukemic stem cells

**Fig 2:**
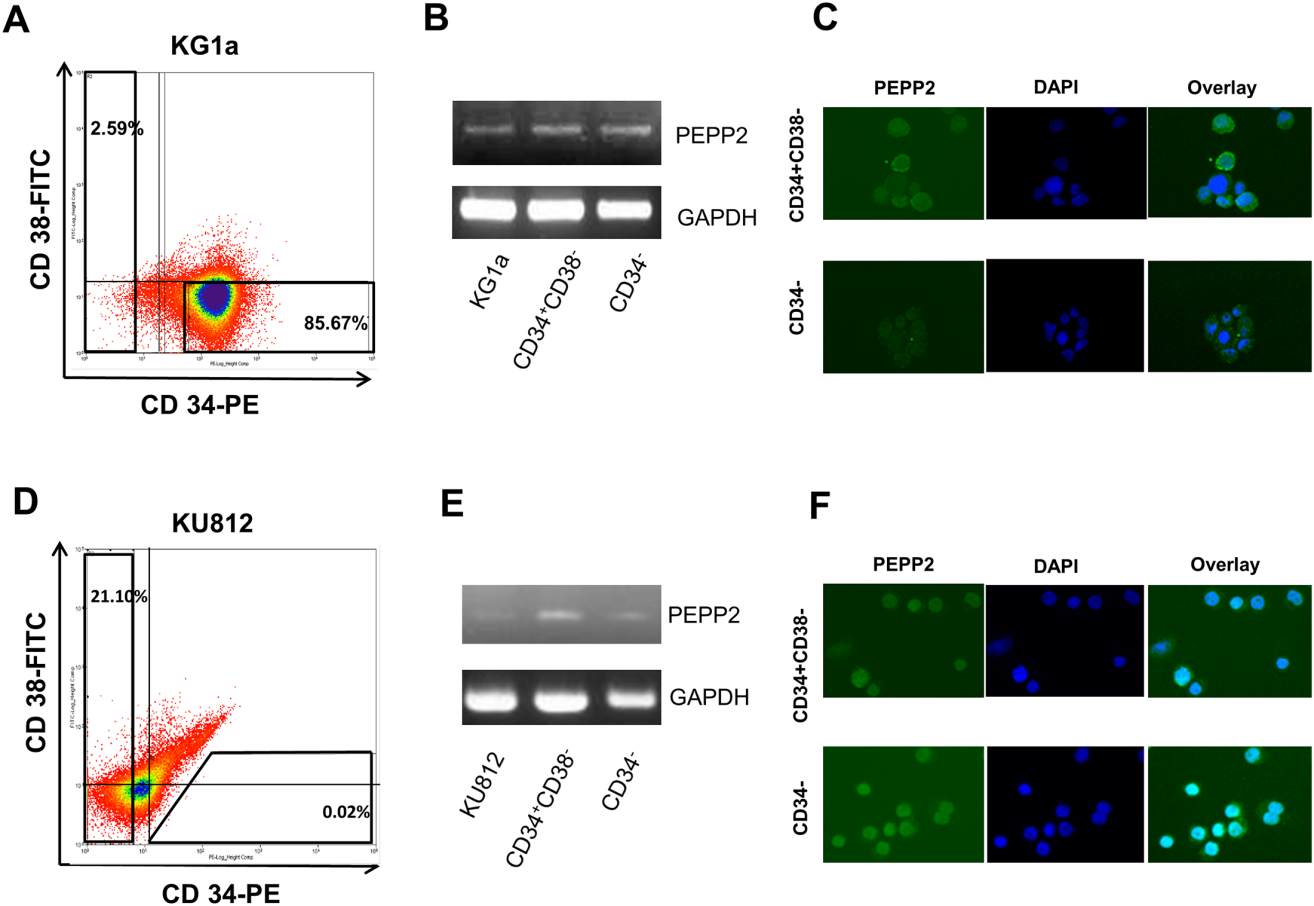
Expression profile of PEPP2 gene in LSCs derived from leukemia cell. AML cell line KG1a (A) and CML cell line KU812 (D) were stained with monoclonal antibodies against CD34 and CD38. CD34^+^38^-^ cells and CD34^-^ cells were obtained by MoFlo cell sorter system. PEPP2 expression in unsorted samples, CD34^+^38^-^ fraction, and CD34^-^ fraction of KG1a were assessed by standard RT-PCR (B,E). PEPP2 protein expression of KG1a (C) and KU812 (F) was detected by immunohistochemistry using anti-PEPP2 monoclonal antibody. These figures show the representative data of 3 independent experiments.

We then examined if the *PEPP2* gene was expressed in LSCs. We sorted the CD34^+^CD38^-^ cells from the AML cell line KG1a (Fig 2A, S2 Fig) and the CML cell line KU812 (Fig 2D, S2 Fig), as LSCs of myelogenous leukemia are considered to be enriched in this fraction, which was also previously reported in leukemia cell lines including KG1a [20, 21]. The sorted CD34^+^38^-^ cells and CD34^-^ cells isolated from both cell lines expressed the *PEPP2* gene (Fig 2B and 2E). Expression of PEPP2 protein was also confirmed by immunohistochemistry (Fig 2C and 2F).

### PEPP2-derived peptide screening

We synthesized 24 PEPP2-derived candidate peptides based on scores predicted by the SYFPEITHI and BIMAS programs (Table 1). We then performed peptide binding and stabilization assays. We first selected 2 peptides, PEPP2^271-279^ (EFGPFPFVI), PEPP2^253-261^ (SLPLPLMLL) which showed high score in stability assay, since peptides recognized by CTL should not only bind to HLA molecule but also stay there for longer time. We also chose PEPP2^140-149^(AFTPIQLQEL), which showed the best binding ability. The three PEPP2 peptides were used for further screening for induction of CTL.

**Table 1:** Screening of HLA-A24:02-restricted epitopes from PEPP2.

**Peptide**	**Sequence**	**BIMAS score**	**SYPFPEITHI score**	**Binding MFI ratio**	**Stability MFI ratio**
**PEPP2**^**194-202**^	**RALMARNML**	**14.400**	**12**	**0.954**	**0.599**
**PEPP2**^**120-128**^	**RPQGAVGGL**	**12.000**	**11**	**0.746**	**0.754**
**PEPP2**^**215-223**^	**AAEAITAPL**	**8.400**	**11**	**0.803**	**0.665**
**PEPP2**^**188-196**^	**KWRRHQRAL**	**8.000**	**11**	**0.772**	**0.714**
**PEPP2**^**141-149**^	**FTPLQLQEL**	**7.920**	**12**	**0.860**	**0.687**
**PEPP2**^**271-279**^	**EFGPFPFVI**	**6.000**	**19**	**1.153**	**1.201**
**PEPP2**^**7-15**^	**CSQYMTSSL**	**6.000**	**12**	**1.103**	**1.041**
**PEPP2**^**253-261**^	**SLPLPLMLL**	**6.000**	**14**	**1.277**	**1.103**
**PEPP2**^**27-35**^	**DMNAMVSL**	**6.000**	**11**	**0.936**	**0.831**
**PEPP2**^**246-254**^	**MPPFPPPSL**	**6.000**	**13**	**0.985**	**0.914**
**PEPP2**^**81-89**^	**GGAGVPGHL**	**5.600**	**13**	**0.852**	**0.785**
**PEPP2**^**117-125**^	**QYSRPQGAV**	**5.000**	**10**	**0.882**	**0.977**
**PEPP2**^**231-239**^	**DYFWDHSHS**	**5.000**	**12**	**0.889**	**0.869**
**PEPP2**^**52-60**^	**QGTAAGEKL**	**4.400**	**13**	**0.820**	**0.938**
**PEPP2**^**145-153**^	**QLQELERIF**	**4.320**	**12**	**0.767**	**1.071**
**PEPP2**^**6-14**^	**QCSQYMTSL**	**4.000**	**10**	**0.724**	**0.614**
**PEPP2**^**25-33**^	**LQDMNAMVL**	**4.000**	**10**	**0.768**	**0.761**
**PEPP2**^**248-256**^	**PFPPPSLPL**	**3.600**	**19**	**1.069**	**0.926**
**PEPP2**^**138-146**^	**VHAFTPLQL**	**0.400**	**13**	**0.992**	**0.748**
**PEPP2**^**144-152**^	**LQLQELERI**	**3.600**	**13**	**0.695**	**0.738**
**PEPP2**^**150-158**^	**ERIFQREQF**	**0.300**	**13**	**0.747**	**0.690**
**PEPP2**^**277-285**^	**FVIVPSFTF**	**3.000**	**13**	**1.181**	**0.928**
**PEPP2**^**140-149**^	**AFTPIQLQEL**	**31.680**	**7**	**1.363**	**0.851**
**PEPP2**^**9-18**^	**QYMTSLLSPA**	**9.000**	**10**	**1.354**	**0.865**
**EBV**	**TYGPVFMCL**	**403.200**	**24**	**1.204**	**1.320**

HLA-A*24:02-binding peptides derived from the PEPP2 protein were predicted using BIMAS and SYFPEITHI. Scores of those algorithms are indicated. HLA-A*24:02-binding peptide derived from EBV was used as a positive control. Mean-fluorescence intensity (MFI) ratio was calculated as shown in Materials and Methods. The average of the two assays is indicated.

### Induction of anti-PEPP2 CTL from healthy donors

**Fig 3:**
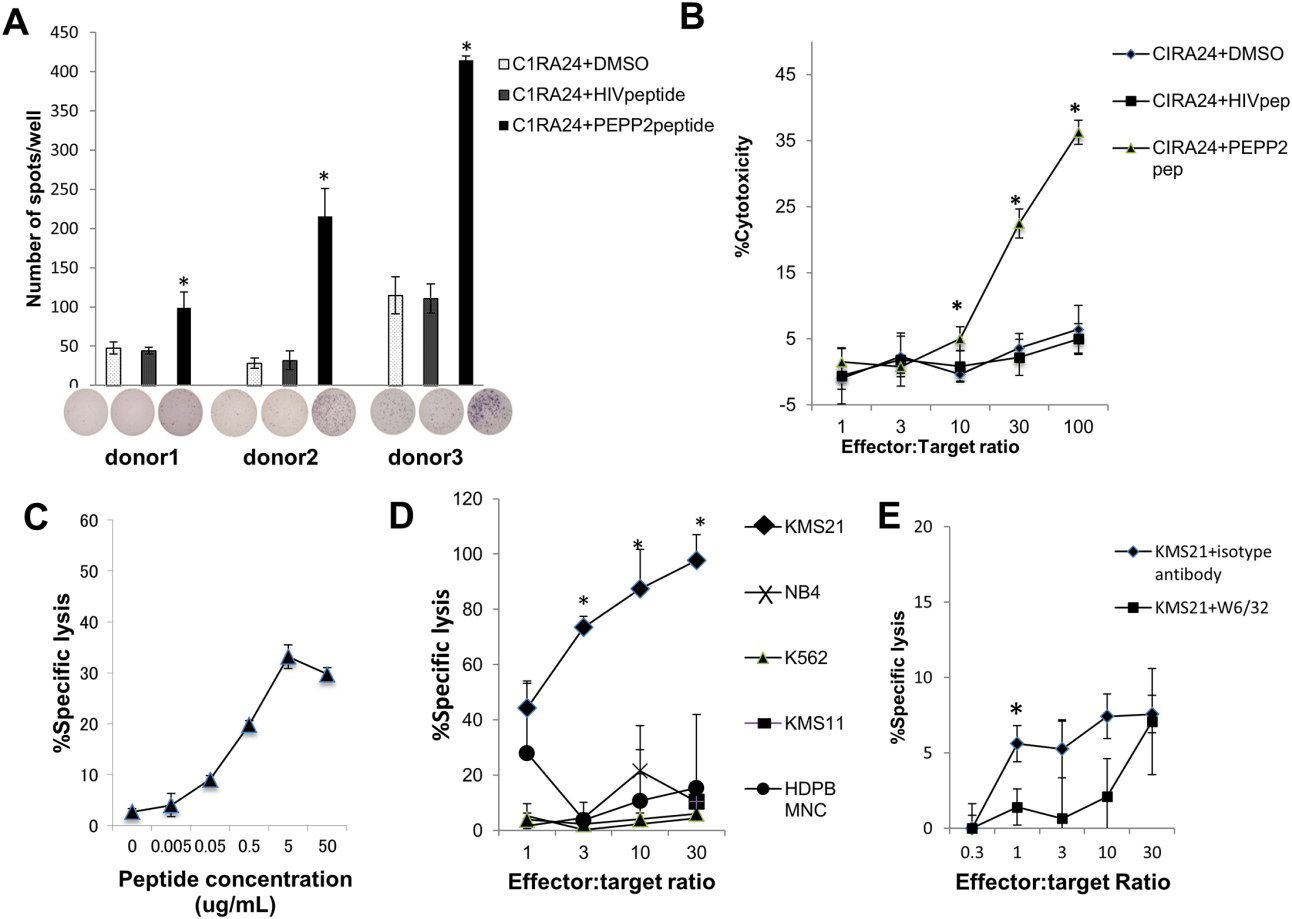
Recognition by PEPP2-specific CTLs. Antigen specificity of PEPP2-specific CTLs was analyzed. (A) IFN-γ secretion by CTLs responding to CIR-A24 cells pulsed with PEPP2^271-279^ or HIV-derived peptide was evaluated by ELISpot assay. The CTLs used in the assays were induced by 3 stimulations and analyzed at the 5th to 7th days after last stimulation.Y-axis indicates the number of spots observed in each well, which contains 3x10^4^ CD8-positive cells. (B) Cytotoxicity of PEPP2-specific CTLs was examined by ^51^Cr release assay. (C) Cytotoxicity of PEPP2-specific CTLs against CIR-A24 cells pulsed with various concentrations of PEPP2^271-279^ was examined at E/T ratio of 30. (D) Cytotoxicity of PEPP2-specific CTLs against cancer cell lines KMS11 (HLA-A*24:02-positive, PEPP2-negative), KMS21 (HLA-A*24:02-positive, PEPP2-positive), NB4 (HLA-A*24:02-negative, PEPP2-positive), and K562 (HLA-A*24:02- negative, PEPP2- positive), and PBMCs from HLA-A*24:02-positive HD was assessed by ^51^Cr release assay. (E) KMS21 cells were pre-treated with anti HLA class I antibody or mouse isotype IgG1 antibody and used for cytotoxicity assay. Experiments were performed in triplicate. *p < 0.05 (Students *t*-test).

Using the PEPP2^271-279^ peptide, we could induce PEPP2-specific CTL from HLA-A*24:02 positive lymphocytes isolated from healthy donors (Fig 3A). We could not induce PEPP2-specific CTL with the PEPP2^253-261^ or PEPP2^140-149^ peptides (data not shown).

The CTLs could lyse C1R-A24 cells only when the target cells were pulsed with PEPP2^271-279^ peptide (Fig 3B). The recognition by CTLs was peptide dose-dependent (Fig 3C). We also examined their cytotoxicity against several cancer cell lines. The CTLs lysed only HLA-A*24:02-positive and PEPP2-positive KMS21 cells (Fig 3D) and failed to recognize HLA-A*24:02-positive PBMNC from HD which expressed low levels of PEPP2 expression (HD7 in Fig 1E). This cytotoxicity was suppressed by addition of anti-HLA class I antibody (Fig 3E).

### Up-regulation of PEPP2 expression by 5-aza-2′-deoxycytidine

**Fig 4:**
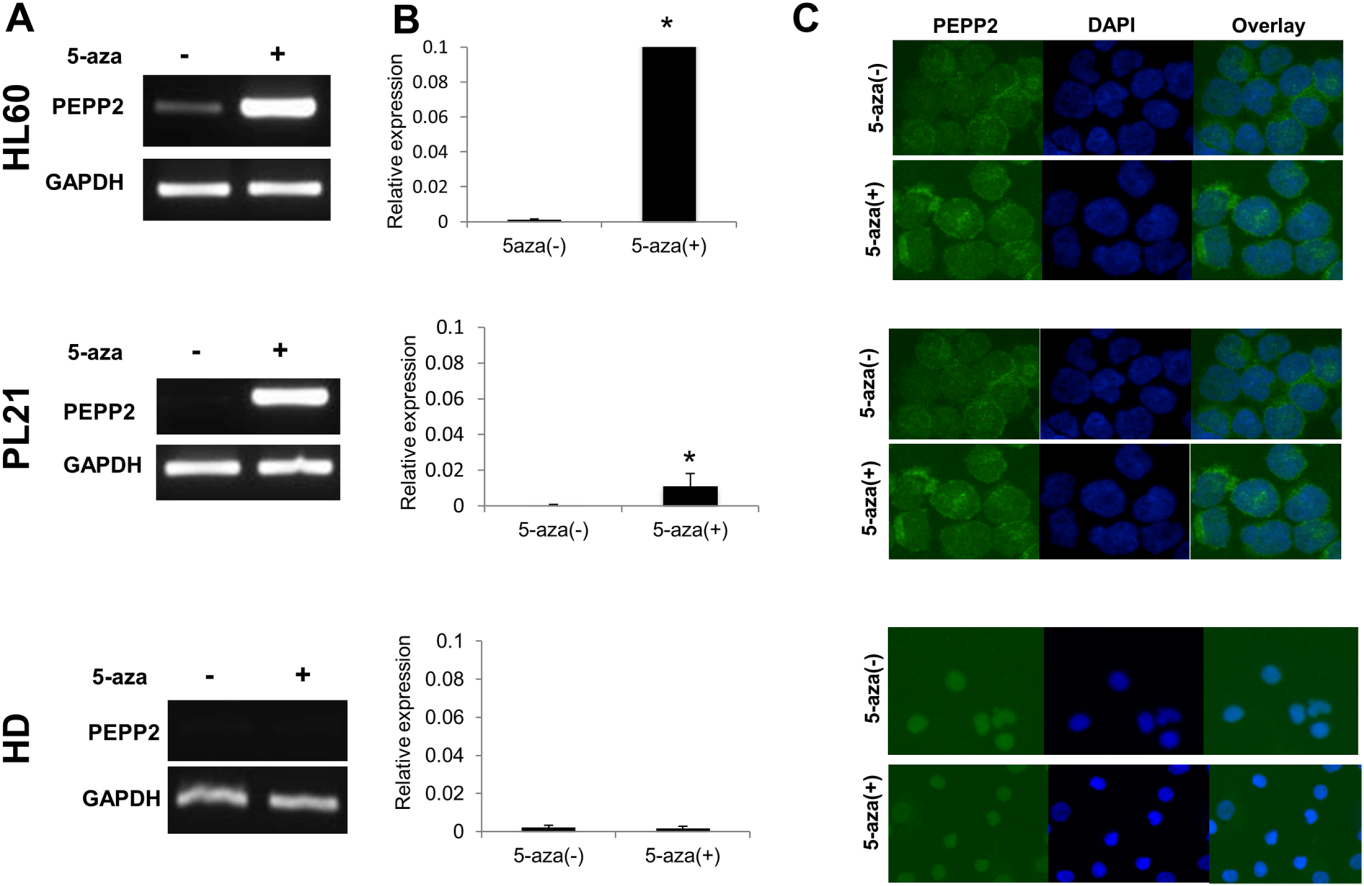
Effects of 5-aza-2′-deoxycytidine on PEPP2 expression. PL21 and HL60 cells, and PBMNCs from HD were incubated with 200 nM of 5-aza-2′-deoxycytidine for 72 hours. RNA was extracted and RT-PCR (A) or quantitative PCR (B) was performed. Cells were also examined for protein expression by immunohistochemistry (C). Quantitative PCR was conducted in triplicate. These figures show the representative data of 2 independent experiments. *p < 0.05 (Students *t*-test).

Gene expression of many CTAs is controlled by methylation of their promoter regions. Therefore, we checked whether PEPP2 expression is up-regulated by 5-aza-2p-deoxycytidine in leukemia cell lines HL60 and PL21, which don’t express *PEPP2* gene. As shown in Fig 4A and 4B, *PEPP2* gene expression was significantly up-regulated after treatment with 5-aza-2′-deoxycytidine. PEPP2 protein expression was also increased in treated cells compared to untreated cells (Fig 4C). In contrast, gene and protein expression did not change after treatment of PBMCs from healthy donor with 5-aza-2′-deoxycytidine.

### Anti-PEPP2 CTLs recognized 5-aza-2P-deoxycytidine -pretreated leukemic cells, but not healthy donor’s PBMNCs

We then examined whether PEPP2-specific CTLs could recognize HLA-A*24:02-positive and PEPP2-negative cancer cells following their treatment with 5-aza-2′-deoxycytidine.

**Fig 5:**
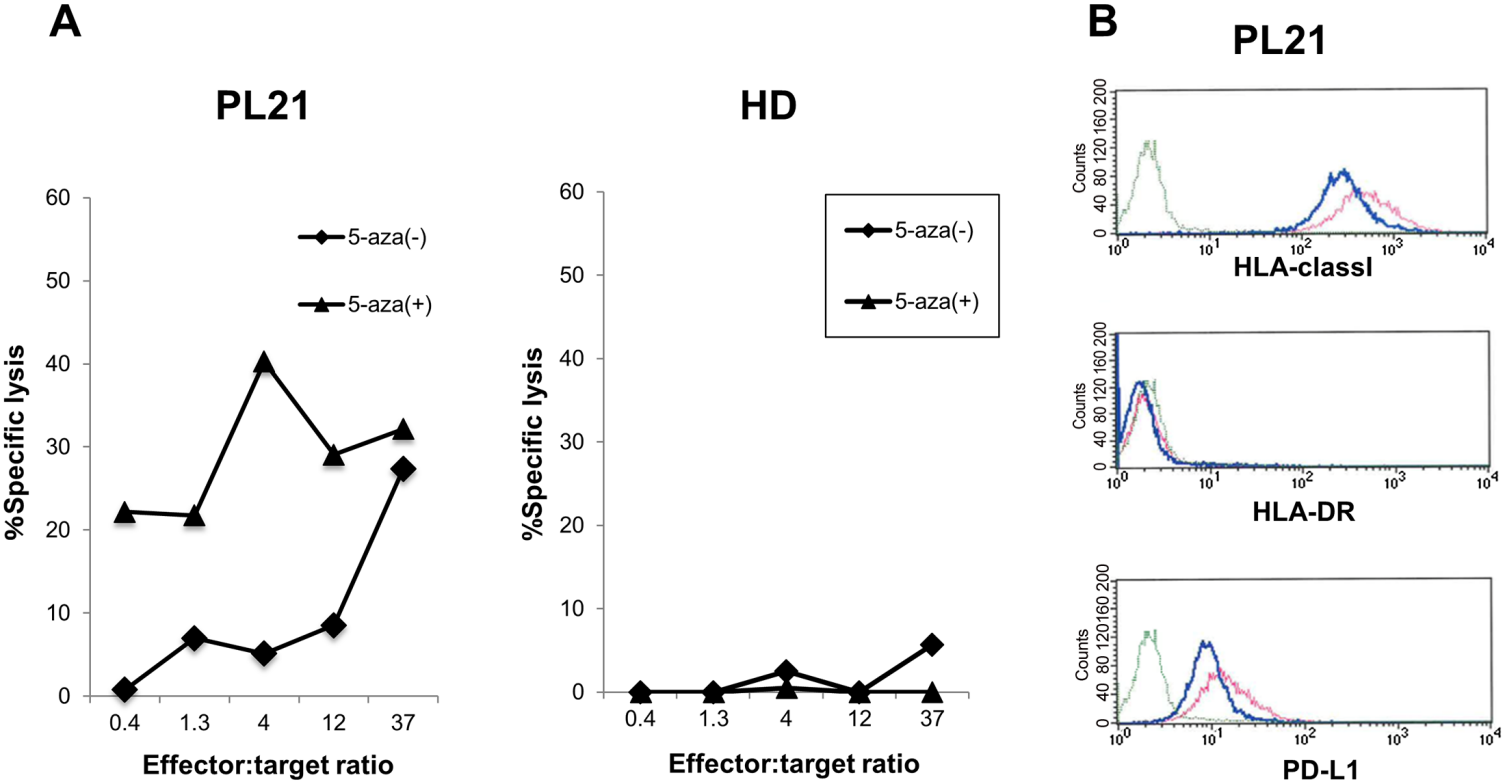
Effects of pretreatment of target cells with 5-aza-2′-deoxycytidine. (A) PL21 cells or PBMNCs from HLA-A*24:02-positive healthy donor were pretreated with 5-aza-2′-deoxycytidine for 72 hours, then evaluated for their sensitivity to lysis by PEPP2-specific CTLs. (B) Expression of HLA class I, HLA-DR, and PD-L1 was assessed in PL21 cells before and after treatment with 5-aza-2′-deoxycytidine. Green line; isotype control. Blue line; pre-treatment. Red line; post-treatment. These figures show the representative data of 2 independent experiments.

As described in Fig 5A the CTLs showed enhanced cytotoxicity against 5-aza-2′-deoxycytidine -treated cells compared with non-treated cells. On the other hand, the CTLs didn’t recognize 5-aza-2′-deoxycytidine -treated normal PBMNCs. We also examined whether the expression of HLA-class I, HLA-DR, and PD-L1 in PL21 cells changed after 5-aza-2′-deoxycytidine treatment. We found that HLA-class I expression was up-regulated. At the same time, PD-L1 expression level was also slightly up-regulated following 5-aza-2′-deoxycytidine treatment (Fig 5B). This up-regulation of PD-L1 was confirmed by quantitative PCR (S3 Fig).

## Discussion

Many CTAs have been reported, however, the number of antigens that can elicit strong immunity and eradicate cancer cells in patients is limited [22].

In this study, we tried to identify the first epitope peptide derived from a homeobox protein, PEPP2. Homeobox genes encode 60-amino acid homeodomains, which are considered to be transcription factors that regulate the developmental process, and sometimes carcinogenesis [23]. Although the target gene or precise function of human PEPP2 is still unknown, Hu et al. [24] reported that Rhox5, a mouse homologue of human PEPP2, could suppress *Unc5c*, or netrin-1 receptor, which is a tumor-suppressor gene with pro-apoptotic function. Li et al. [25] showed that knockdown of Rhox5 in a colon cancer cell line suppressed cell proliferation and migration in mice. There is also a report that transducion of PEPP2 into murine monocytic leukemia cell line enhanced leukemogenesis in irradiated mice [26]. These results indicate an important role of PEPP2 in the development of leukemia and suggest that targeting PEPP2 might be effective in the eradication of leukemia cells.

We first clarified that the *PEPP2* gene is highly expressed in cell lines derived from hematological malignancies and found that especially myeloid leukemia such as APL and CML cells expressed high amount of PEPP2. Then, we also observed its high expression in several BM samples from APL and CML patients, some of which were negative for WT1, suggesting that using both antigens as CTL epitopes would broaden the number of patients who can receive immunotherapy.

On the other hand, *PEPP2* expression in PBMNC or BMMNC from healthy donors was not observed or was extremely low compared to leukemia cells, which suggests that PEPP2 is an ideal target for therapy of leukemia. We didn’t observe high expression of the *PEPP2* gene in other types of cancer cells including brain tumor, lung cancer, and pancreatic cancer, although it has been reported that this gene is expressed in colon cancer and gastric cancer [26], which were not included in our sample set.

Since target antigens expressed by cancer stem cells are better candidates for therapeutic targets, we then examined whether the *PEPP2* gene is expressed in LSCs. We found that the *PEPP2* gene was expressed in both the CD34^+^CD38^-^ fraction and the CD34^-^ fraction in KG1a and KU812 cells. Although its expression level was not restricted to the CD34^+^CD38^-^ fraction, immunotherapy targeting PEPP2 might contribute to eradicating residual LSCs in myeloid leukemia patients.

We also identified a HLA-A*24:02-restricted epitope (PEPP2^271-279^) by screening 24 PEPP2-derived candidate peptides. This epitope is located in the C-terminal domain of the 288 amino acid sequence of PEPP2 and does not overlap with the homeodomain, indicating that this epitope is PEPP2-specific. Anti-PEPP2 CTLs could be consistently induced from PBMNCs of HLA-A*24:02-positive healthy donors. These CTLs recognized epitope peptide-pulsed target cells as well as PEPP2-positive cancer cells in an HLA-A*24:02-restricted manner, indicating that PEPP2^271-279^ is an HLA-A*24:02-restricted epitope specific to PEPP2.

Antigen-specific immunotherapy cannot be applied for patients without antigen expression by their cancer cells. Therefore, induction of target antigens in antigen-negative cancer cells is useful for the treatment of wide variety of cancer patients. Demethylating agents including 5-aza-2′-deoxycytidine is known to increase expression of several CTAs, such as MAGE and NY-ESO1 by epigenetically modulating their gene expression [27]. It has been shown that the expression level of the *Rhox5* gene is up-regulated by epigenetic drugs in mouse cancer cell lines via demethylation of the gene promoter region [28]. Here we evaluated PEPP2 expression in human leukemic cells and normal blood cells after treatment with physiologically tolerable doses of 5-aza-2′-deoxycytidine. Expression level of both the *PEPP2* gene and protein were significantly enhanced in AML cell lines that originally didn’t express PEPP2.

Moreover, PBMNCs from healthy donors failed to express the *PEPP2* gene after treatment with 5-aza-2′-deoxycytidine. In a clinical setting, this phenomenon is extremely important to avoid side effects caused by target expression in normal cells. Coral et al [29] have reported that expression of the MAGE gene family is also up-regulated only in tumor tissue in mouse, however, the precise mechanism causing this difference between cancer cells and normal cells remains to be investigated.

We also revealed that 5-aza-2′-deoxycytidine-treated leukemia cells became sensitive to killing by anti-PEPP2 CTLs. This killing was not a direct effect of the drug on leukemia cells, since viability of leukemia cells was still more than 90% after treatment with the drug (data not shown). In addition, the CTL recognition was not observed with healthy donor’s PBMNCs treated with 5-aza-2′-deoxycytidine, which didn’t express PEPP2, suggesting that this CTL recognition was PEPP2-specific. This finding is consistent with reports using other antigens, such as NY-ESO1 and PRAME [30,31]. In addition, the expression level of HLA class I in leukemia cells was up-regulated after treatment with 5-aza-2′-deoxycytidine, which could facilitate killing of leukemia cells by CTLs. These epigenetic drugs have been approved in many countries and widely used for the treatment of patients with myelodysplastic syndrome. Toor et al. [32] recently reported that administration of azacitidine and lenalidomide followed by autologous lymphocyte infusion could enhance expression of CTAs including MAGEA3 in myeloma patients and antigen-specific CTLs were observed in some subjects. Therefore, the combination of PEPP2-specific immunotherapy with these epigenetic drugs might be an attractive strategy for treatment of leukemia patients. However, we also found that PD-L1, an immune checkpoint molecule, was slightly elevated in 5-aza-2i-deoxycytidine-treated leukemia cells, which might have reduced CTL recognition. This up-regulation of PD-L1 has also been reported in other cancer, such as non-small cell lung cancer or MDS, although the precise mechanism is remained to be determined [33, 34]. Therefore, addition of anti-PD-L1 blockade, which is now widely gaining attention for its immune-modifying ability in cancer treatment, might be necessary for further enhancement of cytotoxic activity against the target cells [35].

In conclusion, we have demonstrated that a homeobox protein, PEPP2, is highly expressed in myeloid leukemia cells and its expression was up-regulated by demethylating agent in a leukemia-specific manner. We also identified a novel HLA-A*24:02-binding epitope, PEPP2^271-279^, which might represent a good target antigen for immunotherapy in therapy-resistant myeloid leukemia patients, especially in combination with a demethylating agent.

## Supporting Information

S1 Fig *PEPP2* gene expression in various normal tissues and cancer cell lines. The expression pattern of the *PEPP2* gene was detected by DNA microarray (A), and *PEPP2* was highly expressed in testis and myelogenous leukemia cell lines (APL, CML). PEPP2 expression level was compared between normal testis and K562 by standard PCR (B) and quantitative PCR (C). Water was used for template as negative control (NC).
(TIF)

S2 Fig Sorting efficacy of LSC in KU812 cells and KG1a cells. CD34^+^CD38^-^ fraction from KU812 (A,B) or KG1a (C,D) was sorted using cell sorter after staining with monoclonal antibodies against CD34 and CD38. Sorted cells were analyzed for its expression of CD34 and CD38.
(TIF)

S3 Fig PD-L1 expression in PL21 cells after treatment with 5-aza-2′-deoxycytidine. PL21 cells were incubated with 200 nM of 5-aza-2′-deoxycytidine for 72 hours. RNA was extracted and quantitative PCR of *PD-L1* gene or *GAPDH* gene was performed. Relative expression was calculated by dividing expression level of PD-L1 by that of GAPDH.
(TIF)
