# Supplementary figures and images for: Identification of Novel HLA-A*24:02-Restricted Epitope Derived from a Homeobox Protein Expressed in Hematological Malignancies
Source: PLoS One. 2016 Jan 19;11(1):e0146371. doi: 10.1371/journal.pone.0146371 (PMC4718592; doi:10.1371/journal.pone.0146371)

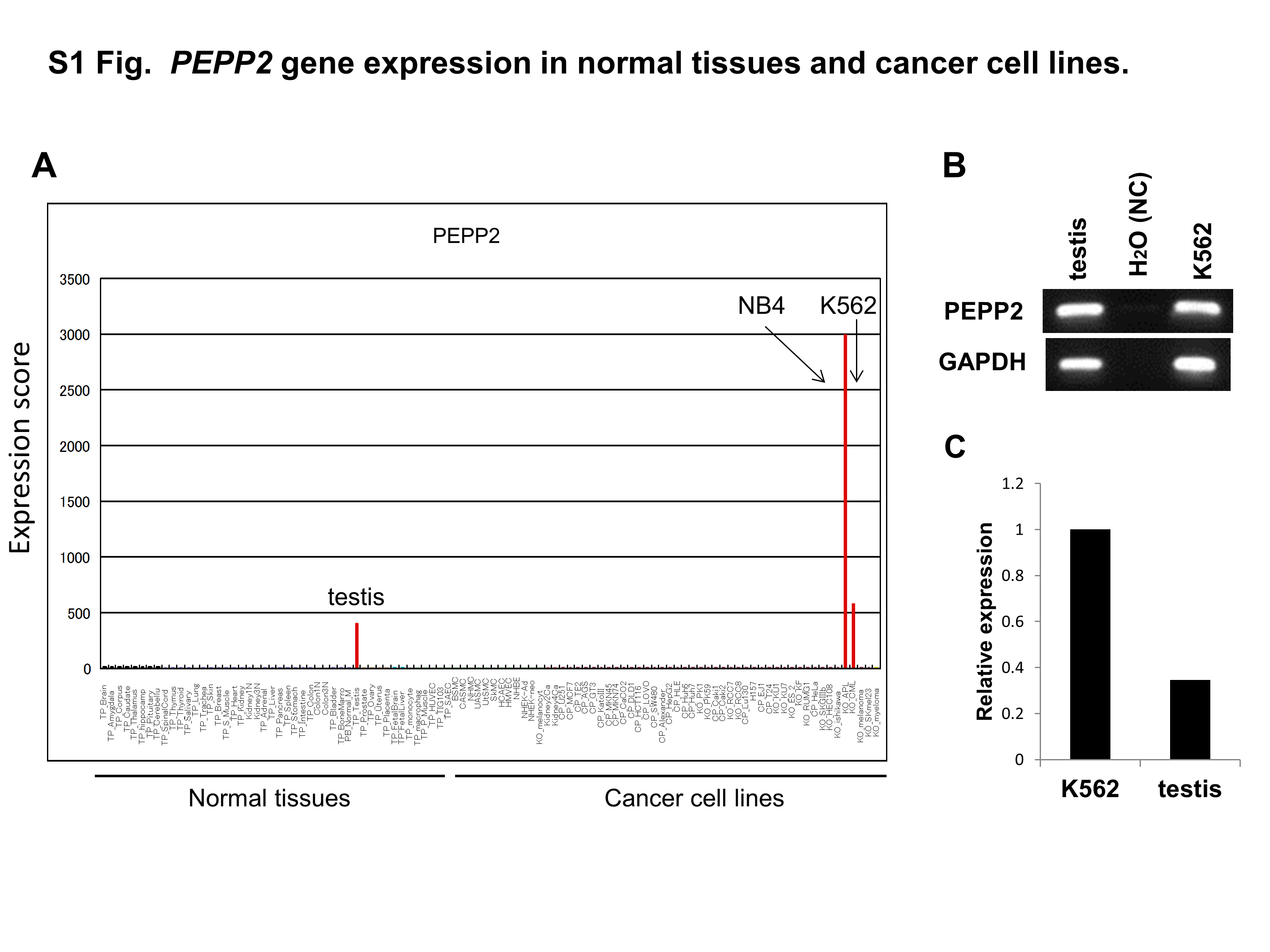

Supplement: S1 Fig — The expression pattern of the PEPP2 gene was detected by DNA microarray (A), and PEPP2 was highly expressed in testis and myelogenous leukemia cell lines (APL, CML). PEPP2 expression level was compared between normal testis and K562 by standard PCR (B) and quantitative PCR (C). Water was used for template as negative control (NC). (TIF) [file pone.0146371.s001.TIF]

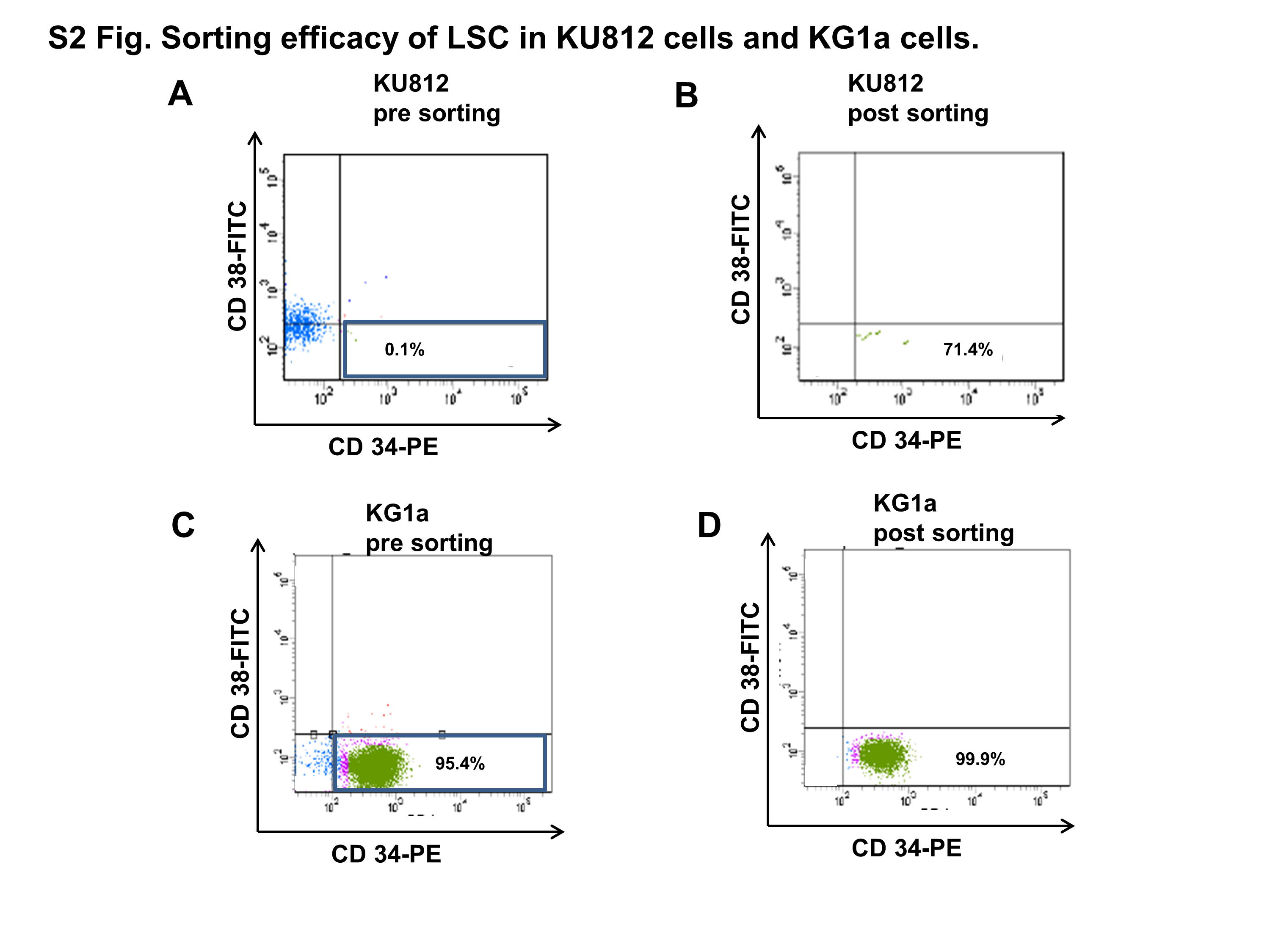

Supplement: S2 Fig — CD34+CD38- fraction from KU812 (A,B) or KG1a (C,D) was sorted using cell sorter after staining with monoclonal antibodies against CD34 and CD38. Sorted cells were analyzed for its expression of CD34 and CD38. (TIF) [file pone.0146371.s002.TIF]

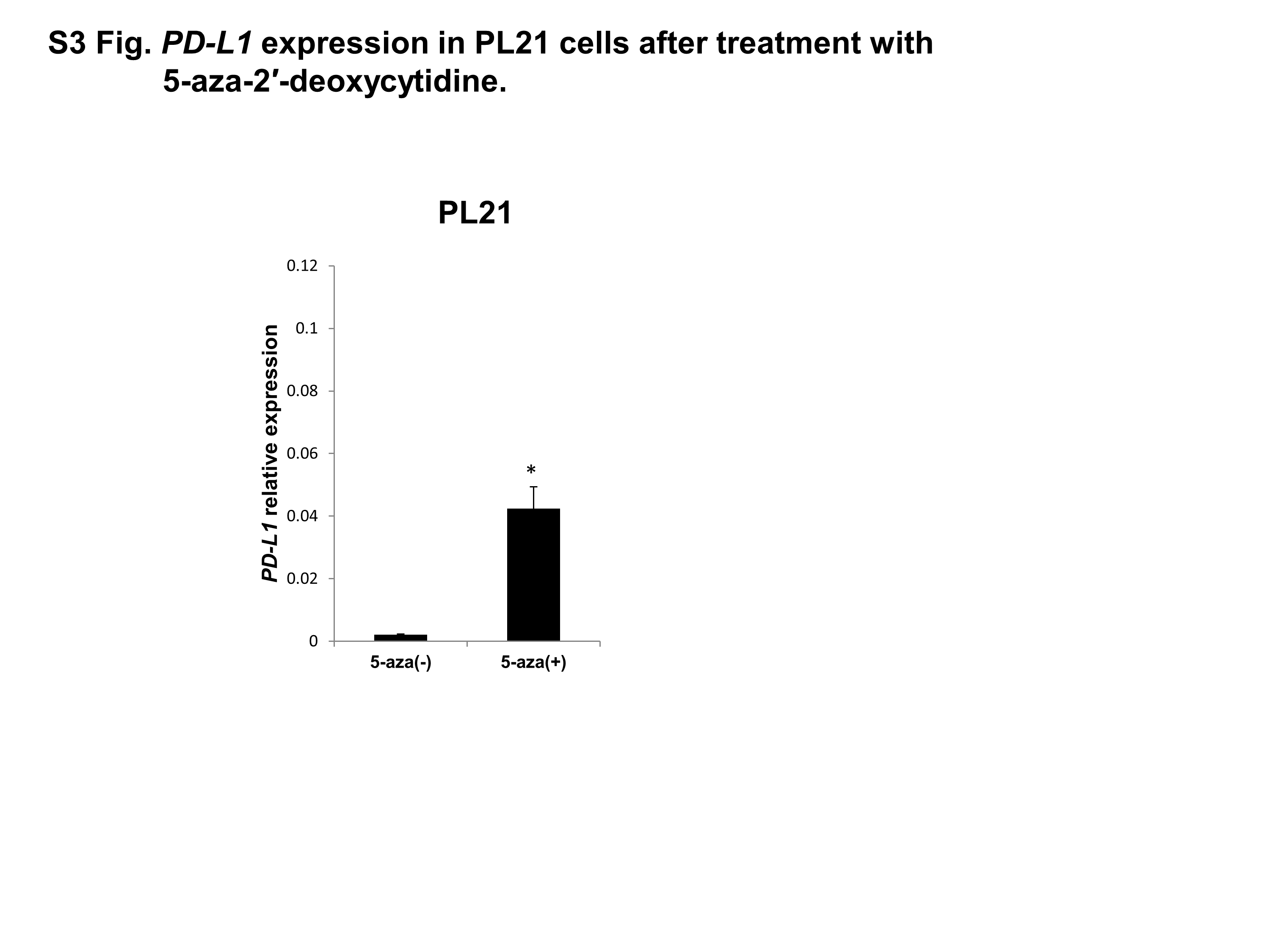

Supplement: S3 Fig — PL21 cells were incubated with 200 nM of 5-aza-2′-deoxycytidine for 72 hours. RNA was extracted and quantitative PCR of PD-L1 gene or GAPDH gene was performed. Relative expression was calculated by dividing expression level of PD-L1 by that of GAPDH. (TIF) [file pone.0146371.s003.TIF]
